# Enhanced lipid metabolism confers the immunosuppressive tumor microenvironment in CD5-positive non-MYC/BCL2 double expressor lymphoma

**DOI:** 10.3389/fonc.2022.885011

**Published:** 2022-10-06

**Authors:** Meng-Ke Liu, Li-Li Cheng, Hong-Mei Yi, Yang He, Xiao Li, Di Fu, Yu-Ting Dai, Hai Fang, Shu Cheng, Peng-Peng Xu, Ying Qian, Yan Feng, Qian Liu, Li Wang, Wei-Li Zhao

**Affiliations:** ^1^ Shanghai Institute of Hematology, State Key Laboratory of Medical Genomics, National Research Center for Translational Medicine at Shanghai, Ruijin Hospital, Shanghai Jiao Tong University School of Medicine, Shanghai, China; ^2^ Department of Pathology, Ruijin Hospital, Shanghai Jiao Tong University School of Medicine, Shanghai, China; ^3^ State Key Laboratory of Microbial Metabolism, School of Life Sciences and Biotechnology, Shanghai Jiao Tong University, Shanghai, China; ^4^ Laboratory of Molecular Pathology, Pôle de Recherches Sino-Français en Science du Vivant et Génomique, Shanghai, China

**Keywords:** diffuse large B cell lymphoma (DLBCL), CD5 positive, non-MYC/BCL2 double expressor, tumor microenvironment, lipid metabolism

## Abstract

Lymphoma cells expressing CD5 (CD5+) confer inferior outcome of diffuse large B-cell lymphoma (DLBCL), especially in non–MYC/BCL2 double expressor (non-DE) patients. In tumor microenvironment, CD5+ non-DE tumor revealed increased proportion of immunosuppressive M2 macrophages and enhanced pathways related to macrophage activation and migration. In accordance to M2 activation, lipid metabolism was upregulated, including fatty acid uptake and fatty acid oxidation, which supplied energy for M2 macrophage polarization and activation. Meanwhile, CD36 expression was upregulated and strongly correlated to the proportion of M2 macrophages in CD5+ non-DE DLBCL. *In vitro*, a DLBCL cell line (LY10) overexpressing CD5 significantly increased M2 proportion in comparison with control when cocultured with peripheral blood mononuclear cells (PBMCs). The addition of metformin significantly decreased the M2 proportion and the CD36 expression level in the coculture systems, indicating that metformin could target altered lipid metabolism and decrease M2 macrophages in DLBCL, especially in CD5+ non-DE lymphoma. In conclusion, enhanced lipid metabolism and M2 macrophage activation contributed to the immunosuppressive tumor microenvironment and could be potential therapeutic targets in CD5+ non-DE DLBCL.

## Introduction

Diffuse large B-cell lymphoma (DLBCL) is the most prevalent type of non-Hodgkin lymphoma and is heterogeneous in clinical and molecular features (1). Although standard immunochemotherapy (R-CHOP regimen: rituximab, cyclophosphamide, doxorubicin, vincristine, and prednisone) has cured about 60% of DLBCL patients, 40% DLBCL patients experience relapse and remain refractory to conventional treatment (2).

Accumulating data have revealed that CD5-positive (CD5+) DLBCL patients fail to benefit from rituximab-based immunochemotherapy and intensive regimens, with the 5-year survival rate as only 40% (3–5). CD5+ DLBCL, a rare disease, accounts for 5%–10% of DLBCL and is defined as an immunohistochemical subtype according to the World Health Organization classification (6). As reported, CD5+ DLBCL is related to an increased serum lactate dehydrogenase level, an advanced Ann Arbor stage, high risk scores of an international prognostic index (IPI), and more frequencies of central nervous system (CNS) relapse (7, 8). Although CD5 is regarded as a poor prognosis biomarker of DLBCL, several studies indicate the heterogeneities of CD5+ DLBCL. Salles et al. reported that CD5 expression over 75% by immunohistochemistry indicated poor outcome (9). Recently, Shen et al. reported that CD5+ double expressor (DE; co-expression of MYC/BCL2) had inferior survival as compared with CD5+ non–double expressor (non-DE) patients (10). Therefore, it is important to explore the genetic alterations in CD5+ DLBCL.

Tumor microenvironment (TME) has been documented to have an essential role in the progression and chemoresistance of DLBCL (11, 12). Immunosuppressive TME accelerates tumor progression through impeding effector T cell and natural killer (NK) cell activation and recruiting immunosuppressive cells such as regulatory T cells, myeloid-derived suppressor cells, and macrophages (13, 14). Tumor-associated macrophages (TAMs) are notorious immune suppressors, supporting tumorigenesis, vascularization, and immunoevasion (15).

Extensive studies have illustrated the profound influence of metabolic reprogramming on TME (16). Immune cells are characterized by different metabolic phenotypes in their proliferation and activation. Activated T and NK cells preferentially use aerobic glycolysis for energy supplement. By contrast, other immunosuppressive cells such as regulatory T cells and myeloid-derived suppressor cells rely on fatty acid oxidation and oxidative phosphorylation for cell function (17). Metabolic changes can interfere the polarization of TAMs to either pro-inflammatory M1 or anti-inflammatory M2. Transcriptional analyses distinguish M1 and M2 in several metabolic pathways. M1 is enriched by glycolysis, glycerophospholipid metabolism, and fructose and mannose metabolism, whereas M2 is enriched by glutamate, purine, arginase, and fatty acid metabolisms (18).

Enhanced fatty acid metabolism is one of the critical metabolic adaptations of M2. Fatty acid transporter (CD36), fatty acid–binding proteins (FABPs), and fatty acid synthase (FASN) are the main essential proteins involved in the M2 lipid metabolism (19). CD36 is strongly expressed on M2 macrophages, contributing to prolonged survival and activation of M2 macrophages (20). Uptake of triacylglycerol substrates *via* CD36 enhances downstream peroxisome proliferator-activated receptor-γ (PPAR-γ) and arginase expression, also following mitochondrial biogenesis and fatty acid oxidation in M2 (21). Stimulation of ox-LDL induces M2 polarization, with a subsequent increase in the expression of arginase-1, interleukin-10 (IL-10), and transforming growth factor-beta (TGF-β), which represent M2 activation (22). Thus, targeting CD36 on M2 provided a possible approach in targeting lipid metabolism to interfere immunosuppressive TME.

Here, we focus on the clinical, genetic, and microenvironment characteristics in CD5+ non-DE DLBCL and further explore the influence of lipid metabolism on the TME.

## Methods

### Patients

**Figure 1:**
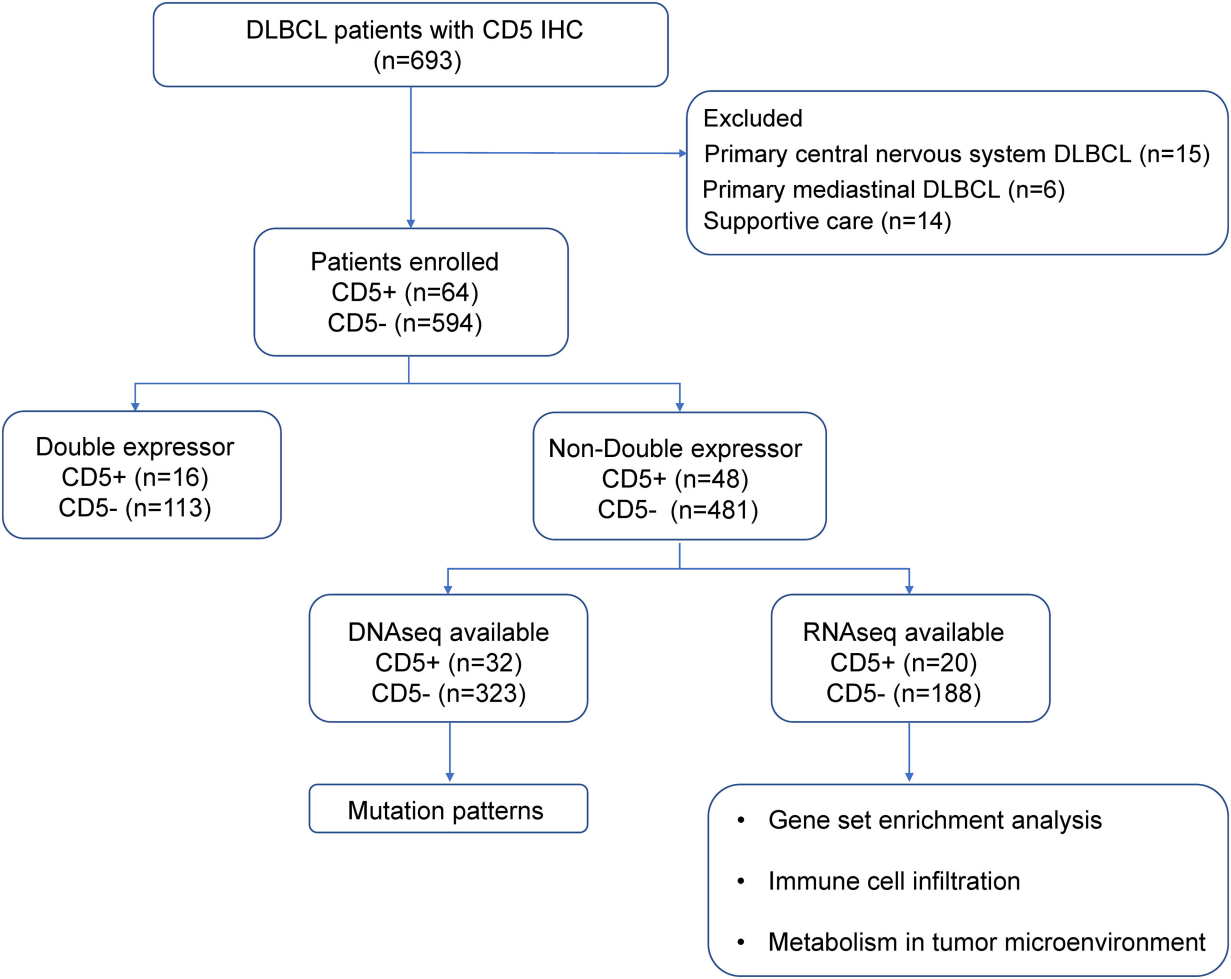
Flowchart describing the procedure of patient selection. DLBCL, diffuse large B-cell lymphoma; IHC, immunohistochemistry.

As shown in the flowchart (**Figure 1**), a total of 658 newly diagnosed DLBCL patients treated in Ruijin Hospital from 2006 to 2019 were included in this study, namely 64 patients with CD5+ and 594 patients negative for CD5 (CD5−). Patients with primary CNS lymphoma, with primary mediastinal lymphoma, or who received supportive care were excluded. All patients were treated with the R-CHOP–based therapy (rituximab, cyclophosphamide, doxorubicin, vincristine, and prednisone) and were included in the survival analysis. There were 529 non-DE DLBCL patients, namely 48 CD5+ non-DE patients and 481 CD5− non-DE patients. Among the 48 CD5+ non-DE patients, eight were recruited in a prospective, single-arm phase II clinical trial (ChiCTR-OIN-17012130) to evaluate the therapeutic effect of metformin as maintenance therapy, in which patients received metformin orally at a dose of 1.0 g, twice a day for 2-year maintenance after complete remission (23). The remaining 40 patients did not receive metformin maintenance therapy. Among 658 patients, 355 had available DNA sequencing, and 208 had available RNA sequencing data.

### Clinical features and pathological data

Clinical data including age, Eastern Cooperative Oncology Group performance status, Ann Arbor stage, serum lactate dehydrogenase, IPI, and sites of extranodal involvement were collected. Immunohistochemistry was performed for CD10, BCL6, MUM1, BCL2, MYC, and CD5 using an indirect immunoperoxidase method on the paraffin sections of tumor tissues according to standard protocols. The histological diagnosis and percentage of positive tumor cells were confirmed by two experienced pathologists. Germinal center B-cell or non–germinal center B-cell origin was classified by Hans algorithm. MYC/BCL2 DE lymphoma was defined as MYC expressed on ≥40% of tumor cells and BCL2 expressed on ≥50% of tumor cells (24).

### DNA sequencing

Genomic DNA from frozen tumor tissues was extracted using a QIAamp DNA Mini Kit (Qiagen, Hilden, Germany); DNA from formalin-fixed paraffin-embedded tumor tissues was extracted using a GeneRead DNA Formalin-Fixed Paraffin-Embedded Tissue Kit (Qiagen). For whole genome sequencing (WGS), sequencing was performed with 150-bp paired-end strategy on an Illumina HiSeq platform in WuXi NextCODE, Shanghai. For whole exome sequencing (WES), exome regions were captured by a SeqCap EZ Human Exome Kit (version 3.0), and sequencing was performed with 150-bp paired-end strategy on a HiSeq 4000 platform in Righton, Shanghai. Burrows–Wheeler Aligner version 0.7.13-r1126 was applied to align read pairs to Human Reference Genome version hg19 (downloaded from UCSC Genome Browser, URLs). Samtools version 1.3 was used to remove PCR duplications and generate chromosomal coordinate-sorted bam files. Genome Analysis Toolkit version 3.4 Haplotype Caller and Genome Analysis Toolkit Unified Genotyper were applied to call single-nucleotide variations (SNVs) and indels. Annotation of SNVs and indels was applied using the UCSC Genome Browser (http://genome.ucsc.edu). The filter of SNVs and indels was based on the following pipelines: (1) germline mutations detected in paired blood samples were excluded, (2) mutations with low frequency (<0.05) were excluded, and (3) mutations included in the single-nucleotide polymorphism database and not reported in COSMIC (the Catalogue of Somatic Mutations in Cancer) version v77 were excluded.

For targeted sequencing, PCR primers were designed by Primer 5.0 software. Multiplexed libraries of tagged amplicons from tumor tissue samples were generated by Shanghai Righton Bio-Pharmaceutical Multiplex–PCR Amplification System.

All data of DNA sequencing including targeted sequencing (n = 178), WES (n = 119), and WGS (n = 58) were collected from our previous report (14).

### RNA sequencing

RNA was extracted from frozen tumor tissue samples with a Trizol and RNeasy Mini Kit (Qiagen) and quantified with NanoDrop. Details of RNA sequencing procedures and RNA sequencing data of 208 patients were all cited from our previous report (14). The raw reads were aligned to human reference genome hg19 with a Burrows‐Wheeler Aligner (v0.7.13‐r1126). Transcript counts table files were generated with HTSeq. Normalization of raw reads and analysis of differentially expressed genes were carried out using R package “limma” (v3.48.1) (25). Gene set enrichment analysis (GSEA) was performed with R package “clusterProfiler” (v4.0.0) based on MSigDB-curated gene sets (c2.cp.keggADD.v7.0.symbols.gmt and c5.bp.v7.0.symbols.gmt) (26). Metabolic signature scores were calculated using single-sample GSEA through R package “GSVA” (1.40.1); gene sets of lipid metabolism were acquired from the KEGG database. A CIBERSORT deconvolution algorithm (v 1.03) was applied in the calculation of immune cell populations based on its reference list. A total of 22 immune cell phenotypes were included in the analysis according to the signatures of bulk RNA-sequencing profile.

### Cells and reagents

The B-lymphoma cell line OCI-LY-10 was provided by Huang CX. DB and THP-1 cells were purchased from the American Type Culture Collection. PBMCs from a healthy donor were obtained *via* Ficoll–Paque density gradient centrifugation. Cells were cultivated in IMDM or RPMI 1640, with the addition of 10% heat-inactivated fetal bovine serum and 1% penicillin/streptomycin (15140122, Gibco, Carlsbad, CA, USA) under a humidified atmosphere containing 95% air–5% CO_2_ at 37 °C. Metformin was obtained from Selleck (Houston, TX, USA). Sulfosuccinimidyl oleate (SSO) was obtained from MedChemExpress (Shanghai, China).

### Cell transfection

CD5 overexpression cells or negative control cells were constructed using lentiviral transfection. A purified plasmid MSCV-IRES-EGFP-CD5 (GV358) retroviral vector or control vector was transfected into package HEK-293T cells. Lentiviral particles were collected from the supernatant of HEK-293T cells and condensed to a viral concentration of ~2 × 10^8^ transducing units/ml. Then, LY-10 cells or DB cells were cultivated, with the addition of lentiviral particles for 72 h in IMDM or RPMI 1640 containing polybrene (8 μg/ml). Flow cytometry was applied to select stably transduced clones expressing EGFP fluorescence protein.

### Flow cytometry

PBMCs were cocultured with CD5 overexpressed OCI-LY-10 or vector OCI-LY-10 in IMDM and CD5 overexpressed DB in RPMI 1640 for 72 h. The following anti-human antibodies were used: APC anti-CD14 (555397, BD Biosciences), PE-CY7 anti-CD68 (565595, BD Biosciences), PE anti-CD163 (556018, BD Biosciences), and Percp-Cy5.5 anti-CD36 (336224, Biolegend). Flow cytometry was applied on a FACS Calibur cytometer (BD Biosciences) and analyzed by FlowJo software (v10.4).

### Generation of M2 macrophages from THP-1 cell line

To induce M2 macrophages, 320 nM phorbol myristate acetate was added to THP-1 cells for 6 h, followed by phorbol myristate acetate plus 20 ng/ml of IL-4 and IL-13 for 18 h (27).

### Oil red O staining

Oil red O staining was applied using a modified Lipid Staining Kit (C0158S, Beyotime) following its staining protocol. Cells were fixed with 10% formalin for 10 min and washed with phosphate-buffered saline twice. Staining wash buffer was added for 20 s and then removed. Then, cells were stained with oil red O solution for 20–30 min, and then the stain solution was removed. Oil red O staining was observed under a microscope.

### Quantitative real-time PCR

Total RNA was extracted from M2 cells using a Trizol reagent and reverse-transcribed into cDNA using a HiScript III RT SuperMix for qPCR with a gDNA wiper (R323-01, Vazyme). Quantitative real-time PCR was performed with a ChamQ Universal SYBR qPCR Master Mix (Q711-02, Vazyme) using ABI ViiA7 (Applied Biosystems, Bedford). Forward primer of CD36: 5′-GGCTGTGACCGGAACTGTG-3′; reverse primer: 5′-AGGTCTCCAACTGGCATTAGAA-3′; forward primer of FASN: 5′-CCGAGACACTCGTGGGCTA-3′; reverse primer: 5′-CTTCAGCAGGACATTGATGCC′; forward primer of FABP5: 5′-TGAAGGAGCTAGGAGTGGGAA-3′; reverse primer: 5′-TGCACCATCTGTAAAGTTGCAG′. Relative expression was calculated and described using ^2−ΔΔ^CT.

### Statistical analysis

The clinical characteristics of patients were assessed using Pearson’s χ^2^ test or Fisher’s exact test. Differences of immune cell populations and normalized gene expression in two groups were carried out using unpaired *t*-test or Mann–Whitney *U* test. Progression-free survival (PFS) was determined as the time from the date of diagnosis to the date when the disease progression of relapse was recognized or the date of last follow-up. Overall survival (OS) was determined as the time from the date of diagnosis to the date of death or the date of last follow-up. Survival functions were analyzed using the Kaplan–Meier method and compared by the log-rank test. Statistical significance was defined as p < 0.05. All statistical analyses were performed using R software (version 4.1.0; http://www.R-project.org) and Statistical Package for the Social Sciences (SPSS, 25.0) software (SPSS, Inc., Chicago, IL, USA).

## Results

### Clinical characteristics and survival

**Figure 2:**
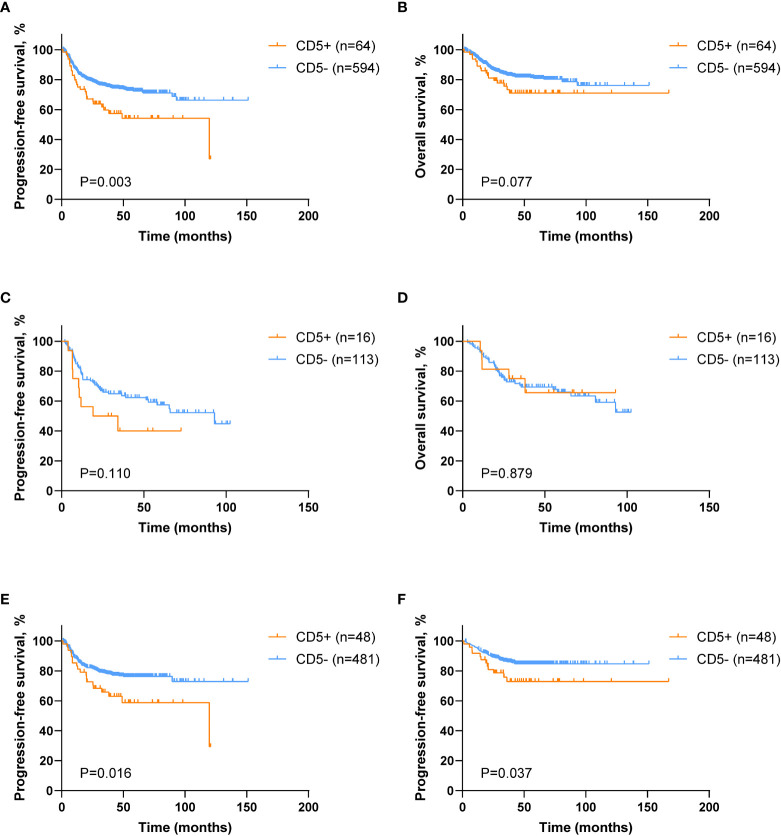
Survival analysis of DLBCL patients according to CD5 positivity. **(A, B)** Kaplan–Meier survival curves of progression-free survival (PFS) and overall survival (OS) comparing CD5+ (n = 64) and CD5− (n = 594) DLBCL patients. **(C, D)** Kaplan–Meier survival curves of PFS and OS in CD5+ (n = 16) and CD5− (n = 113) double expressor (DE) DLBCL patients. **(E, F)** Kaplan–Meier survival curves of PFS and OS in CD5+ (n = 48) and CD5− (n = 481) non–double expressor (non-DE) DLBCL patients.

The median follow-up of 658 patients was 41.3 months, and the 5-year PFS and the 5-year OS were 70.6% and 80.0%, respectively. However, CD5+ DLBCL patients had poor 5-year PFS (54.2% vs. 72.4%, p = 0.003; **Figure 2A**), but without statistical difference in the 5-year OS (71.0% vs. 80.9%, p = 0.077; **Figure 2B**) when compared with CD5− DLBCL patients. Among the DE patients, no significant difference was observed between CD5+ and CD5− patients in terms of PFS (5-year PFS 40.0% vs. 59.3%, p = 0.110; **Figure 2C**) and OS (5-year OS 65.6% vs. 65.9%, p = 0.879; **Figure 2D**). Nevertheless, CD5+ non-DE patients had poor outcome, as compared with CD5− non-DE patients: 5-year PFS 58.8% vs. 76.3%, p = 0.016 (**Figure 2E**) and 5-year OS 72.9% vs. 84.7%, p = 0.037 (**Figure 2F**).

Based on these findings that CD5+ had prognostic indications in non-DE DLBCL, further clinical, genetic, and microenvironment profiles were analyzed in non-DE patients. Among non-DE, CD5+ patients had advanced Ann Arbor stage (III–IV) (p = 0.021), high-risk IPI (p = 0.010), more frequent bone marrow involvement (p = 0.018), and high CNS relapse (p = 0.040; **Table 1**).

**Table 1:** Clinical characteristics of CD5+ and CD5− non-DE DLBCL patients. .

Characteristic	CD5+ (n = 48)	2.1.1.1 CD5− (n = 481)	p
**Median age, years (range)**	57 (25–90)	58 (16–90)	
**Gender**			0.079
Men Women	32 (66.7%) 16 (33.3%)	257 (53.4%) 224 (46.6%)	
**Ann Arbor, n (%)**			0.021
I–II	21 (43.7%)	293 (60.9%)	
III–IV	27 (56.3%)	188 (39.1%)	
**LDH, n (%)**			0.241
> Normal	26 (54.2%)	218 (45.3%)	
≤ Normal	22 (45.8%)	263 (54.7%)	
**ECOG, n (%)**			0.330
< 2	43 (89.6%)	449 (93.3%)	
≧ 2	5 (10.4%)	32 (6.7%)	
**IPI, n (%)**			0.010
0–2	27 (56.2%)	355 (73.8%)	
3–5	21 (43.8%)	126 (26.2%)	
**Age > 60 years, n (%)**	19 (39.6%)	202 (42%)	0.747
**Extranodal sites, n (%)**			0.231
≧ 2	15 (31.3%)	113 (23.5%)	
< 2	33 (68.7%)	368 (76.5%)	
**Cell of origin**			0.443
Non-GCB	30 (62.5%)	373 (56.8%)	
GCB	18 (37.5%)	208 (43.2%)	
**Response**			0.413
CR	38 (79.2%)	403 (83.8%)	
Non-CR	10 (20.8%)	78 (16.2%)	
**Bone marrow involvement, n (%)**	8 (16.7%)	31 (6.4%)	0.018
**CNS relapse, n (%)**	3 (6.3%)	6 (1.3%)	0.040

DLBCL, diffuse large B-cell lymphoma; LDH, lactate dehydrogenase; ECOG, Eastern Cooperative Oncology Group; IPI, international prognostic index; GCB, germinal center B-cell; CR, complete remission; CNS, central nervous system.

### Mutation patterns

**Figure 3:**
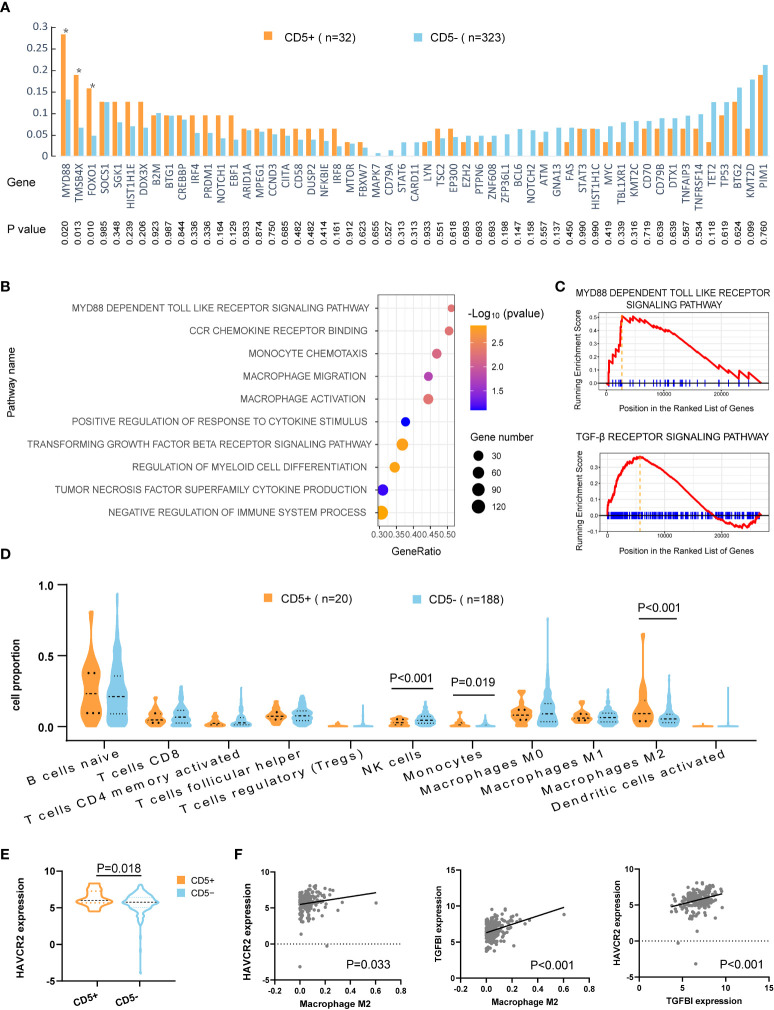
Mutation patterns and tumor microenvironment of CD5+ non-DE patients. **(A)** Genetic mutations of 55 genes in CD5+ (n = 32) and CD5− (n = 323) non-DE patients. P values are listed in the lower part of the graph. **(B)** Upregulated gene ontology (GO) pathways in CD5+ non-DE (n = 20) compared with CD5− non-DE (n = 188) patients. The color gradient represents −log_10_ (p value) of GO pathways, the point size indicates the gene numbers involved in each gene set, and the position of the *x*-axis indicates the gene ratio of genes enriched in each gene set. **(C)** Enrichment plot of MYD88-dependent Toll-like receptor signaling pathway and TGF-β receptor signaling pathway. **(D)** Proportion of immune cells in the tumor microenvironment of CD5+ non-DE (n = 20) and CD5− non-DE (n = 188) patients. **(E)** Normalized expression of HAVCR2 (TIM3) comparing CD5+ non-DE (n = 20) and CD5− non-DE (n = 188) patients. **(F)** Correlation between M2 macrophage proportion and normalized expression of HAVCR2 or TGFBI, and correlation between normalized expression of HAVCR2 and TGFBI. P values were listed in each plot.

Oncogenic mutations related to DLBCL progression were detected in WES, WGS, and targeted sequencing. Mutation patterns in non-DE patients were displayed in **Figure 3A**. The mutation frequencies of MYD88 (28.1% vs. 13.0%, p = 0.020), FOXO1 (18.8% vs. 6.5%, p = 0.013), and TMSB4X (15.6% vs. 4.6%, p = 0.010) were significantly higher in CD5+ patients than in CD5− patients.

### Gene expression analysis and tumor microenvironment analysis

Gene ontology analysis revealed the activation of multiple pathways, including the MYD88-dependent Toll-like receptor pathway, monocyte- and macrophage-related pathways (monocyte chemotaxis, macrophage migration, macrophage activation, positive regulation of response to cytokine stimulus, regulation of myeloid cell differentiation, tumor necrosis factor superfamily cytokine production, and CCR chemokine receptor binding), and pathways related to inhibit immune responses (negative regulation of immune system process, TGF-β receptor signaling pathway), in CD5+ non-DE patients (**Figures 3B,C**). Following analysis of an immune cell proportion in TME was carried out through CIBERSORT. As displayed in **Figure 3D**, an elevated proportion of monocytes (p = 0.019), M2 macrophage (p < 0.001), and a decreased proportion of NK cells were observed (p < 0.001) in CD5+ non-DE when compared with CD5− non-DE patients. Meanwhile, the normalized expression level of HAVCR2 (also called TIM3) was significantly increased in CD5+ than CD5− non-DE patients (p = 0.018) (**Figure 3E**). Moreover, the normalized expression levels of HAVCR2 (p = 0.033) and TGFBI (p < 0.001) were positively correlated with the M2 proportion in TME, and the normalized expression of HAVCR2 was positively correlated with TGFBI (p < 0.001) (**Figure 3F**).

### Variation in lipid metabolism in RNA sequencing

**Figure 4:**
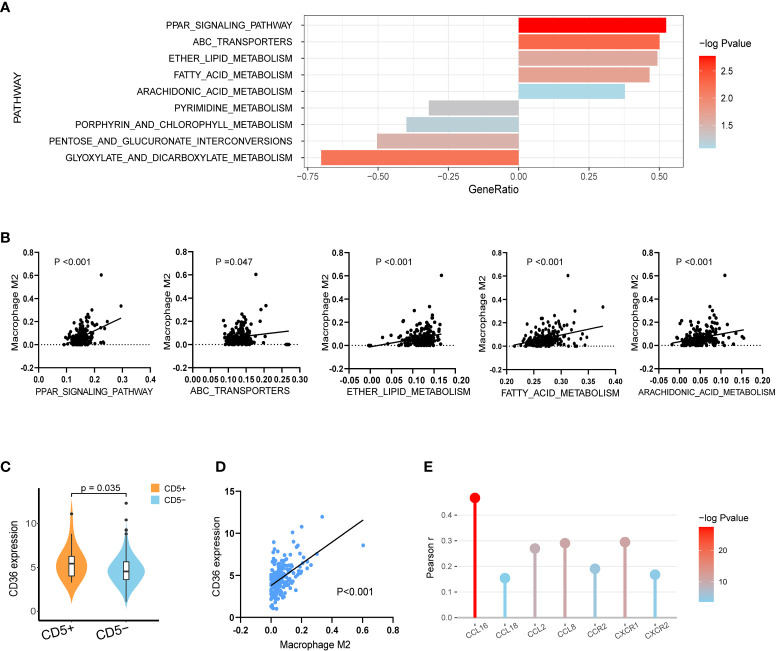
Transcriptional analysis of lipid metabolism alterations in CD5+ non-DE patients. **(A)** The most variable metabolic process upregulated in CD5+ non-DE (n = 20) patients (gene ratio above 0.00) or CD5− non-DE (n = 188) patients (gene ratio below 0.00) based on Kyoto Encyclopedia of Genes and Genomes (KEGG) pathways. **(B)** Correlation between M2 proportions and signature scores of PPAR signaling pathway, ATP-binding cassette transporters pathway, ether lipid metabolism, fatty acid metabolism pathway, and arachidonic acid metabolism pathway in non-DE patients (n = 208). **(C)** Comparison of normalized CD36 expression between CD5+ non-DE (n = 20) and CD5− non-DE (n = 188) patients. **(D)** Correlation between M2 proportion and normalized CD36 expression. P value was labeled in the graph. **(E)** Correlation between normalized expression of CD36 and expression of chemokines or chemokine receptors including CCL16, CCL18, CCL2, CCL8, CCR2, CXCR1, and CXCR2. The color gradient represents −log_10_ (p value), and the *y*-axis represents Pearson *r*.

Regarding the variation in metabolic pathways between CD5+ and CD5− non-DE DLBCL, as revealed by GSEA, upregulation of lipid metabolic pathways, including the PPAR signaling pathway, the ATP-binding cassette transporter pathway, ether lipid metabolism, the fatty acid metabolism pathway, and arachidonic acid metabolism, was observed in CD5+ patients. However, the metabolic pathway enriched in CD5− patients included pyrimidine metabolism, porphyrin and chlorophyll metabolism, pentose and glucuronate interconversions, and glyoxylate and dicarboxylate metabolism (**Figure 4A**). Further comparing the correlation between metabolic pathways and immune cell proportion, all these upregulated metabolism pathways in CD5+ non-DE patients were positively correlated with the M2 proportion (**Figure 4B**). Among differentially expressed genes, the normalized expression level of CD36 was significantly higher in CD5+ than CD5− non-DE patients (p = 0.035; **Figure 4C**) and positively correlated with the M2 proportion in TME (p < 0.001; **Figure 4D**). Additionally, the expression level of CD36 was also positively correlated with the expressions of multiple chemokines and chemokine receptors including CCL16, CCL18, CCL2, CCL8, CCR2, CXCR1, and CXCR2 (**Figure 4E**).

### Metformin attenuated CD36 expression and lipid metabolism

**Figure 5:**
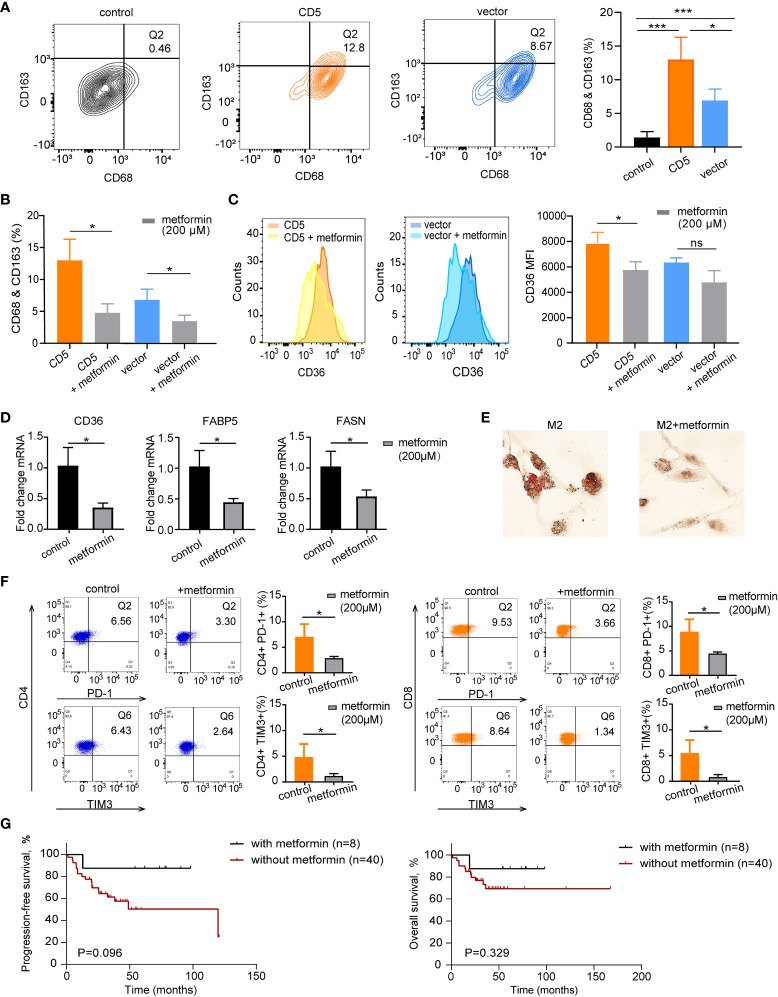
The therapeutic impacts of metformin on M2 lipid metabolism and CD36 expression. **(A)** Flow cytometry analysis of M2 macrophages (Q2) proportion based on co-expression of CD68 and CD163 in PBMC control, coculture of PBMC and LY-10/CD5, and coculture of PBMC and LY-10/vector for 72 h **(B)** Bar plot of M2 proportion in PBMC cocultured with LY-10/CD5 or LY-10/vector with or without 200 μM metformin treatment for 72 h, summarized as mean ± SD (n = 3). **(C)** Flow cytometry analysis of CD36 expression on M2 macrophages comparing the counts and median fluorescence intensity (MFI) of CD36 on M2 in PBMC cocultured with LY-10/CD5 or LY-10/vector with or without 200 μM metformin treatment. **(D)** The normalized mRNA expression of CD36, FABP5, and FASN in THP-1–induced M2 cells with or without 200 μM metformin treatment. **(E)** Oil red O staining of lipid droplets in THP-1–induced M2 cells with or without 200 μM metformin treatment. **(F)** Expression of immune checkpoint PD-1 and TIM3 on CD4 and CD8 T cells after LY-10/CD5 cocultured with PBMC with or without 200 μM metformin treatment for 72 h **(G)** Kaplan–Meier survival curves of PFS and OS in CD5+ non-DE patients with (n = 8) or without (n = 40) metformin maintenance. * represented significant difference of P<0.05.

After PBMC cocultured with CD5-overexpressed (LY-10/CD5) or vector-transfected (LY-10/vector) cells at the ratio of 5:1 for 72 h, flow cytometry was applied to calculate the proportion of M2 and CD36 expression on M2. As shown in **Figure 5A**, the coculture with LY-10/CD5 or LY-10/vector increased the M2 proportion (13.0% ± 3.3%, p = 0.004 or 6.9% ± 1.7%, p = 0.007) in PBMC as compared with PBMC alone (1.4% ± 0.9%). Of note, the M2 proportion was significantly high in the LY-10/CD5 coculture system than in the LY-10/vector coculture system (p = 0.047; **Figure 5A**). Metformin treatment (200 μM) significantly decreased the proportion of M2 in both coculture systems, from 13.0% ± 3.3% to 4.8% ± 1.4% (p = 0.017) in the LY-10/CD5 coculture system and from 6.9% ± 1.7% to 3.6% ± 0.9% (p = 0.039) in the LY-10/vector coculture system (**Figure 5B**). Further exploring the efficacy of metformin on the expression of CD36, flow cytometry analysis revealed that, after treatment with metformin, the CD36 expression level on M2 was significantly downregulated in the LY-10/CD5 coculture system (p = 0.025) than the LY-10/vector coculture system (p = 0.052; **Figure 5C**). PBMCs cocultured with DB/CD5 cells were also treated with 200 μM metformin for 72 h, M2 cells decreased from 10.8% ± 2.1% to 6.3% ± 1.5% (p = 0.043), and CD36 expression on M2 cells also decreased significantly (p = 0.018) (**Supplementary Figure S1**). M2 cells induced from THP-1 cells were treated with 200 μM metformin for 72 h. The mRNA expression levels of CD36, FABP5, and FASN were significantly decreased after metformin treatment (p = 0.019, p = 0.021, and p = 0.037, respectively) (**Figure 5D**). Also, oil red O staining showed reduced lipid droplets accumulation after metformin treatment (**Figure 5E**).

### Metformin decreased immune checkpoint expression

In the LY10/CD5 and PBMC coculture system, after the addition of metformin (200 μM) for 72 h, the expressions of PD-1 on both CD4 T cells (from 7.0% ± 2.6% to 2.8% ± 0.4%, p = 0.048) and CD8 T cells (from 8.8% ± 2.6% to 4.4% ± 0.4%, p = 0.044) decreased significantly. Meanwhile, TIM3 expression decreased from 5.4% ± 2.6% to 0.8% ± 0.5% on CD8 T cells (p = 0.038) and decreased from 5.0% ± 2.3% to 1.1% ± 0.5% (p = 0.046) on CD4 T cells (**Figure 5F**).

Consistent with the *in vitro* results that metformin targeted immunosuppressive TME, CD5+ non-DE patients with metformin maintenance tended to have longer PFS and OS than those without metformin maintenance (5-year PFS, 87.5% vs. 50.3%, p = 0.096, and 5-year OS, 87.5% vs. 69.3%, p = 0.329; **Figure 5G**).

### CD36 inhibitor interfered lipid metabolism and immune checkpoint expression

**Figure 6:**
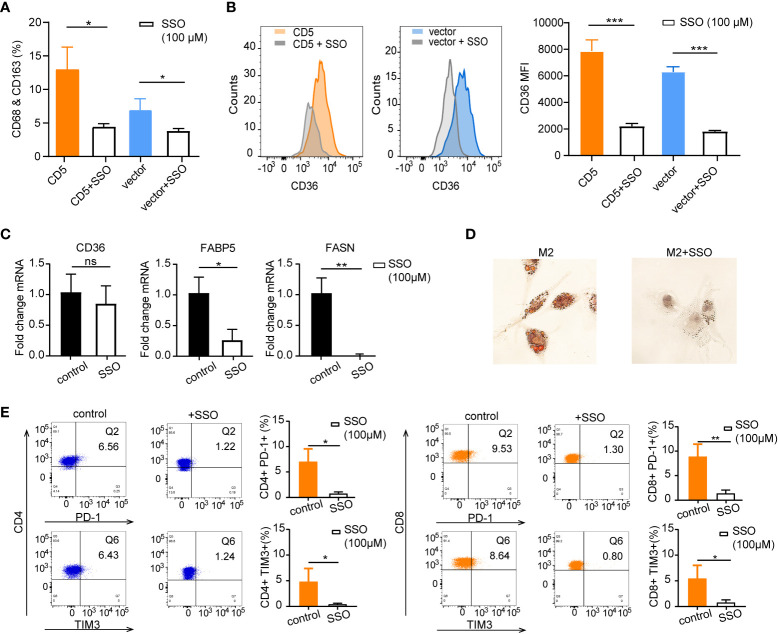
The therapeutic impacts of sulfosuccinimidyl oleate (SSO) on M2 lipid metabolism and CD36 expression. **(A)** Bar plot of M2 proportion in PBMC cocultured with LY-10/CD5 or LY-10/vector with or without 100 μM SSO treatment for 72 h, summarized as mean ± SD (n = 3). **(B)** Flow cytometry analysis of CD36 expression on M2 macrophages comparing the counts and median fluorescence intensity (MFI) of CD36 on M2 in PBMC cocultured with LY-10/CD5 or LY-10/vector with or without 100 μM SSO treatment. **(C)** The normalized mRNA expression of CD36, FABP5, and FASN in THP-1–induced M2 cells with or without 100 μM SSO treatment. **(D)** Oil red O staining of lipid droplets in THP-1–induced M2 cells with or without 100 μM SSO treatment. **(E)** Expression of immune checkpoint PD-1 and TIM3 on CD4 and CD8 T cells after LY-10/CD5 cocultured with PBMC with or without 100 μM metformin treatment for 72 h. * represented significant difference of P<0.05; ** represented significant difference of P<0.01; *** represented significant difference of P<0.001. ns, no significance.

To further explore the impact of CD36 inhibition on lipid metabolism in the coculture system, irreversible CD36 inhibitor SSO (100 μM) was applied in PBMC cocultured with LY-10/CD5 or LY-10/vector for 72 h; flow cytometry was used for the quantification of the M2 proportion and surface CD36 proportion. After SSO treatment, M2 proportion was significantly decreased from 13.0% ± 3.3% to 4.4% ± 0.5% (p = 0.012) in the LY-10/CD5 coculture system and decreased from 6.9% ± 1.7% to 3.8% ± 0.4% (p = 0.035) in the LY-10/vector coculture system (**Figure 6A**), as well as the reduction of surface CD36 proportion on M2 (p < 0.001) (**Figure 6B**). However, SSO treatment did not affect the mRNA expression level of CD36 but significantly decreased the mRNA level of FABP5 (p = 0.014) and FASN (p = 0.002) in M2 (**Figure 6C**). Consequently, the accumulation of lipid droplets was significantly inhibited in M2 upon SSO treatment (**Figure 6D**).

Comparable with the results of metformin treatment, blockage of CD36 with SSO in the coculture system (LY10/CD5 and PBMC) significantly decreased the immune checkpoint expression on CD4 T cells (PD-1: from 7.0% ± 2.6% to 0.7% ± 0.4%, p = 0.013; TIM3: from 5.0% ± 2.3% to 0.4% ± 0.2%, p = 0.026) and CD8 T cells (PD-1: from 8.8% ± 2.6% to 1.3% ± 0.7%, p = 0.009; TIM3: from 5.4% ± 2.6% to 0.8% ± 0.6%, p = 0.037) (**Figure 6E**).

## Discussion

CD5+ DLBCL accounts for approximately 10% of DLBCL, which is commonly reported with inferior prognosis and higher relapse probability of CNS. Our study revealed that CD5 positivity conferred inferior outcome in non-DE patients, but not in DE patients, indicating that CD5+ should be paid more attention among non-DE patients.

In our cohort, MYD88 was the most frequently mutated in CD5+ non-DE DLBCL, consistent with the previous results that MYD88 mutation is supposed to be a genetic feature of CD5+ DLBCL (28, 29). MYD88 is an adaptor protein, receives stimulation from Toll-like and interleukin receptors, and drives the activation of the nuclear factor-κB pathway (30). Interestingly, it has recently been demonstrated that DLBCL with mutant MYD88 exhibited with a macrophage-activating secretion profile through exhibiting elevated CCL2 expression, followed by an increase in macrophage-derived TGF-β1 secretion (31). Meanwhile, higher frequencies of FOXO1 mutation and TMSB4X mutation were also detected in CD5+ non-DE DBLCL. FOXO1 mutation is an unfavorable prognostic factor in DLBCL (32). TMSB4X is regulated by TGF-β and facilitated tumor metastasis in melanoma (33).

Comparable to MYD88 mutation, according to RNA sequencing data, significant upregulation of macrophage activation pathways and infiltration of M2 macrophages were presented in CD5+ non-DE DLBCL, indicating the crucial role of immunosuppressive cells in this subgroup. TGF-β–induced protein (TGFBI) is predominantly secreted by CD163-positive macrophages, which contributes to the suppressive TME (34). In concordance with the increased proportion of M2 in non-DE CD5+ DLBCL, the expression of immune checkpoint TIM3 (HAVCR2) was positively correlated with the proportion of M2 in our study. TIM3 expression on TAMs is conditionally fostered by TGF-β and enhances macrophage activation to promote tumor progression (35). On the other hand, the fraction of NK cells was lower in the TME of CD5+ non-DE DLBCL, which also supports the immunosuppressive TME condition.

Moreover, comparable with the activation and infiltration of macrophages in TME, the activation of macrophage-related metabolism pathways such as the fatty acid metabolism pathway (36) and the PPAR signaling pathway (37) and the regulation of the lipid metabolic pathway were significantly increased in CD5+ non-DE DLBCL. Fatty acid uptake is initialized through CD36 internalization and contributes to M2 polarization (19). Fatty acid metabolism not only supports energy for M2 macrophages (38) but also impairs the antitumor function of NK cells (39) and CD8+ T cells (40). In our cohort of patients, the expression of CD36 was significantly elevated in CD5+ non-DE lymphoma than its CD5− counterpart, consistent with the previous findings that CD36 is one of the top variant genes that distinguished CD5+ from CD5− DLBCL (41, 42).

Metformin, a traditional medicine for the regulation of glucose metabolism, has additional functions on lipid metabolisms. Lipid biogenesis can be downregulated by metformin treatment, which targets sterol regulatory element-binding protein-1c in fatty acid synthesis (43). The expression of scavenger receptor CD36 can also be attenuated by metformin and thus reduces triglyceride contents in cells (44, 45). As displayed in our results, metformin significantly decreased the M2 proportion and inhibited the expression of CD36, FABP5, and FASN, leading to the reduction of lipid droplets in M2, which indicated that lipid metabolism in M2 cells was interfered by metformin. Interestingly, immune checkpoints (PD-1 and TIM3) expression on T cells was downregulated, suggesting that metformin treatment might overcome the immunosuppressive TME in CD5+ non-DE DLBCL. Metformin maintenance therapy did not achieve significantly higher PFS in CD5+ non-DE DLBCL patients; this might be due to the limited number of patients and the short duration of follow-up.

Taken together, in non-DE DLBCL, CD5+ lymphoma represented an increased proportion of M2 and immunosuppressive TME through reprogrammed lipid metabolism and conferred poor prognosis of patients upon R-CHOP treatment. Metformin could target dysregulated fatty acid metabolism through inhibiting CD36 expression on M2 and M2 polarization, rendering the clinical rationale of metformin treatment in CD5+ non-DE DLBCL.

## Data availability statement

The datasets presented in this study can be found in online repositories. The names of the repository/repositories and accession number(s) can be found below: https://www.biosino.org/node/project/detail/OEP001143.

## Ethics statement

The studies involving human participants were reviewed and approved by Shanghai Ruijin Hospital Ethics Board. Written informed consent to participate in this study was provided by the participants’ legal guardian/next of kin.

## Author contributions

W-LZ, LW, P-PX, and SC designed and supervised the study. M-KL, H-MY, YH, and YQ collected clinical data and carried out statistical analysis. M-KL, L-LC, and DF performed biological experiments and made the figures. Y-TD, HF, YF, and QL provided technical support for bioinformatic analysis, and W-LZ, LW, and M-KL drafted the manuscript. All authors contributed to the article and approved the submitted version.

## Funding

This study was supported by the National Natural Science Foundation of China (82170178, 82130004, 81830007, and 82070204), the Shanghai Municipal Education Commission Gaofeng Clinical Medicine (20152206 and 20152208), the Multicenter Clinical Research Project by Shanghai Jiao Tong University School of Medicine (DLY201601), the Clinical Research Plan of Shanghai Hospital Development Center (SHDC2020CR1032B), the Chang Jiang Scholars Program, the Samuel Waxman Cancer Research Foundation, and the Foundation of National Facility for Translational Medicine (Shanghai, TMSK-2020-115).

## Acknowledgments

We appreciate all the efforts of doctors in enrolling patients and collecting clinical data and all the patients involved who allow us to analyze their data.

## Conflict of interest

The authors declare that the research was conducted in the absence of any commercial or financial relationships that could be construed as a potential conflict of interest.

## Publisher’s note

All claims expressed in this article are solely those of the authors and do not necessarily represent those of their affiliated organizations, or those of the publisher, the editors and the reviewers. Any product that may be evaluated in this article, or claim that may be made by its manufacturer, is not guaranteed or endorsed by the publisher.

## Supplementary material

Supplementary Figure 1 The therapeutic impacts of metformin and SSO on M2 proportion and CD36 expression in DB cell lines. **(A)** Flow cytometry analysis of M2 macrophages (Q2) proportion based on co-expression of CD68 and CD163 in PBMCs cocultured with DB/CD5, with or without metformin/SSO treatment for 72 h. Bar plot of M2 proportion in PBMC cocultured with DB/CD5 with or without metformin/SSO treatment for 72 h, summarized as mean ± SD (n = 3). **(B)** Flow cytometry analysis of CD36 expression on M2 macrophages comparing the counts and median fluorescence intensity (MFI) of CD36 on M2 in PBMC cocultured with DB/CD5 with or without metformin/SSO treatment.
